# Fluorometric Index for Sensing Oil in the Sea Environment

**DOI:** 10.3390/s17061276

**Published:** 2017-06-02

**Authors:** Emilia Baszanowska, Zbigniew Otremba

**Affiliations:** Physics Department, Gdynia Maritime University, 81-225 Gdynia, Poland; eba@am.gdynia.pl

**Keywords:** seawater, oil sensing, excitation-emission spectra, fluorometric index

## Abstract

Excitation-emission matrix spectroscopy (EEMS) was applied to determine the fluorometric index (FI) as a parameter indicating the presence of a source of oil pollution in a specific area of the sea. Seawater from the Polish coast (the Baltic Sea) and the same water combined with various amounts of crude oil extracted from the Baltic Sea shelf (*Petrobaltic*-type oil) were used in this study. The FI values were calculated for excitation and emission wavelengths found at the maximal peak, taking into account the natural seawater and the seawater artificially contaminated (for an oil-to-water ratio range of 0.5 × 10^−6^ − 500 × 10^−6^). The wavelength configurations (Ex/Em) (225/355 and 225/340) for the FI index were applied. It was found that, independent of the amount of oil, the FI achieves a higher value for natural seawater than for seawater that has had contact with oil. These results provide the basis to design a sensor signaling the appearance of oil in a defined sea area.

## 1. Introduction

Searching for an efficient tool for oil detection in natural seawater is important for monitoring environmental concerns. Especially the catastrophic oil spill in the Gulf of Mexico in 2010, various sensors have been tested to detect the most harmful oil substances—polycyclic hydrocarbon compounds (PAHs) [1,2]—as well as indicators confirming the presence of oil substances and their influence on natural seawater constituents [3,4]. Despite this, many questions about fluorometric sensor applicability to detect oil (such as wavelength configuration or detection limits) still remain unanswered [2]. However, the information described in numerous papers concerns the challenge of determining the concentration of oily substances in the depths of the sea. There are even sensors produced for monitoring the concentration of oil that may appear in the water after dispersing from a spill on the water’s surface [5]. The intention of the authors of this work is to search for a way to detect oil, even if it appears in the water column or on the sea surface in a place other than the point of sampling or the location of the oil sensor. This is a different approach in the detection of oil by determining the concentration of hydrocarbons in the water. The idea is to make it possible to detect the spillage even before the oil is clearly noticeable (it can be a unintentional or illegal discharge from a ship, a leak from a pipeline or mining equipment, or a seepage from wrecks on the seabed). The Baltic Sea is particularly vulnerable to oil spills due to heavy traffic and other technical activities [6].

Introductory studies on the fluorescence of seawater that has been in contact with oil [7] indicated that the fluorescence properties of seawater may change in the vicinity of oil residing in the marine environment. This is a consequence of the presence of certain oil components leached to water masses, which by diffusion can reach the water sampling point or stationary detector. Oils—petroleum and its derivatives (fuels, lubricate oils)—constitute a highly complex mixture of hydrocarbons, inter alia aromatics [8,9] responsible for the ability to fluoresce, which is particularly noticeable in the ultraviolet range. Oils, when dissolved in non-fluorescing solvents (e.g., n-hexane), produce a characteristic fluorescence spectra, which enable recognizing the type of oil [10,11]. An analogous effect was found for oil-in-water emulsion [12,13], as well as the abovementioned effect of water in contact with oil. However, although the solubility of oil in water is low [14], the presence of a petroleum substance in seawater is significant enough that it is manifested in the fluorescence spectra. Unfortunately, peaks in the fluorescence spectra of natural seawater partially overlap peaks which appear in seawater contacted with oil. Thus, this represents a serious impediment to identifying the oil factor in the fluorescence spectrum. In this paper, the results of excitation-emission fluorescence matrix spectroscopy (EEMS) are reported regarding the possibility of recognizing the presence of oil in the vicinity of a sampling station. This situation occurs in cases when oil, which is invisible to an above-water observer, appears as a sunken spot on the seabed or oil-in-water emulsion in the water column. EEMS were applied for natural seawater and the same seawater intentionally contaminated with various amounts of oil in order to track the main peaks. In conclusion, we propose a method to determine the previously defined fluorometric index (FI), taking into account both natural seawater and seawater polluted by oil, as a value extracted from the excitation-emission spectra (EEMs) for oil located in the vicinity of the sampling station.

## 2. Materials and Methods

### 2.1. Seawater Sampling

Seawater samples were taken at the seaside near Gdynia (Gulf of Gdansk in the Southern Baltic Sea, Poland). Seawater samples were collected at the end of a pier promenade from a 1 m depth into 1 L glass bottles, twice in June 2015. This sampling station was selected due to its location near the Gdynia-Orlowo Cliff—a nature conservation area.

### 2.2. Contamination

**Figure 1:**
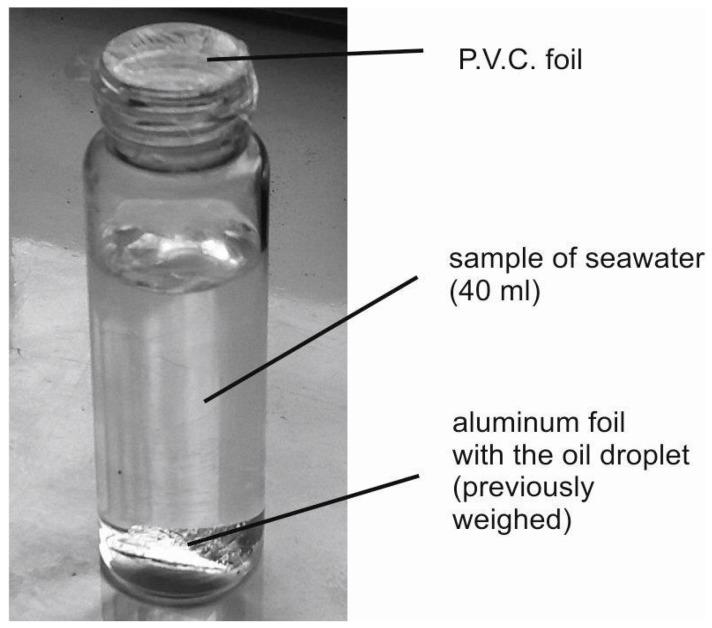
Method of preparing the samples of artificially contaminated seawater (right).

As a material for the laboratory contamination of seawater, crude oil extracted from the Baltic Sea shelf (*Petrobaltic*-type) was used. Small amounts of oil placed on a slice of aluminum foil were weighed and inserted into a seawater sample (Figure 1) to reach the desired oil-to-water ratio. Six samples polluted by oil with an oil-to-water ratio (r_o/w_) from the range of 0.5 × 10^−6^ to 500 × 10^−6^ were prepared. Natural seawater was exposed to the added oil for one day.

### 2.3. Measurement and Apparatus

The excitation-emission spectra were determined using a Hitachi F-7000 FL spectrofluorometer in a 1 × 1 cm quartz cuvette. The following measurement parameters were applied: excitation wavelength from 200 nm to 480 nm with excitation sampling interval 5 nm, emission wavelength from 260 nm to 700 nm with emission sampling interval 5 nm, excitation slit 10 nm, emission slit 10 nm, integration time 0.5 s, and photomultiplier tube voltage 400 V.

First, the EEMs for the natural seawater were determined. Next, the EEMs of seawater exposed to *Petrobaltic* crude oil with various oil concentrations were measured. Measurements for all samples were performed at a stabilized temperature of 20 °C. Rayleigh scattering to yield a digital matrix of the EEMs was removed (if the excitation wavelength and emission wavelengths were equal and the emission wavelength was two times higher than the excitation wavelength).

## 3. Results

### 3.1. EEMs for Original and Contaminated Seawater

**Figure 2:**
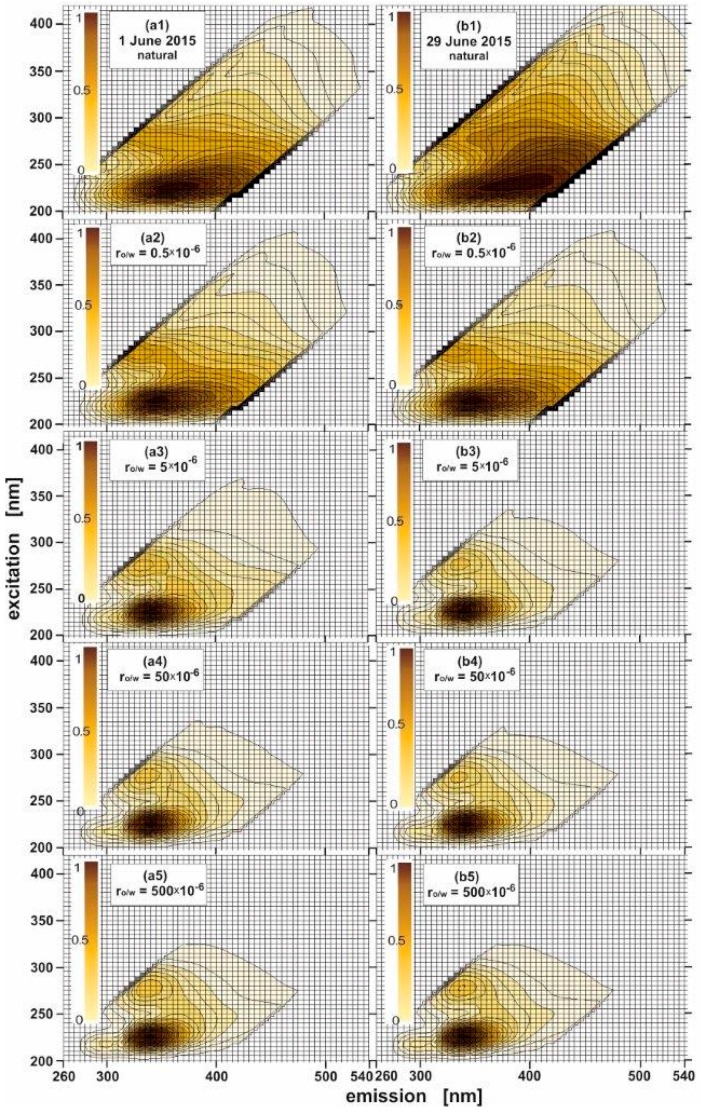
Excitation-emission spectra of natural seawater sampled twice in June 2015: 1 June 2015 (**a1**) and 29 June 2015 (**b1**) and the same water artificially contaminated with various degrees with oil (**a2**–**a5** for 1 June 2015 and **b2**–**b5** for 29 June 2015, respectively). Each spectrum is normalized to its maximal peak (expressed by scale in the color legend for values from 0 to 1).

EEM spectra for natural seawater samples and the same seawater samples combined with oil were determined. Figure 2 presents the EEMs for the seawater sampled in June 2015, day 1 (Figure 2(a1)) and day 29 (Figure 2(b1)) and the same seawater samples polluted by oil for several oil-to-water ratios: 0.5 × 10^−6^, 5 × 10^−6^, 50 × 10^−6^ and 500 × 10^−6^. The EEM spectra of natural seawater presented in Figure 2 indicate the presence of the main peak in the UV-range positioned at an excitation wavelength from 200 nm to 280 nm (centered at 225 nm) corresponding to an emission wavelength from 300 nm to 450 nm centered at 365 nm for a sample from 1 June, and centered at 390 nm for a sample from 29 June. The detected peaks are well-linked to the tryptophan-like seawater component [15,16].

The changes in the shape of the EEMs of seawater can be observed only if the seawater has been contaminated with a small amount of oil which is displayed exactly for the oil-to-water ratio of 0.5 × 10^−6^ in Figure 2(a1,b1). Moreover, when the oil-to-water ratio in seawater increases, the changes in the shape of fluorescence spectra regarding the natural seawater and seawater polluted by oil are clearly visible. Figure 2(a2–a5,b2–b5) present the evolution of the EEM spectra when the oil-to-water ratio increases, starting from 0.5 × 10^−6^ to 500 × 10^−6^. In Figure 2(a2–a5,b2–b5) it is clearly visible that the presence of oil is evidenced in the EEMs by the presence of other excitation-emission peaks. Two specific peaks can be determined for a given oil-to-water ratio range: the major fluorescent peak located at an excitation wavelength from 200 to 260 nm (centered at 225 nm) corresponding to the emission wavelength from 310 nm to 410 nm (centered at 340 nm) described by Ex_max_/Em_max_ = 225/340 nm and the second peak located at an excitation wavelength from 260 nm to 300 nm (centered at 275 nm), corresponding to an emission wavelength position from 310 nm to 360 nm (centered at 335) described by Ex_max_/Em_max_ = 275/335 nm.

**Figure 3:**
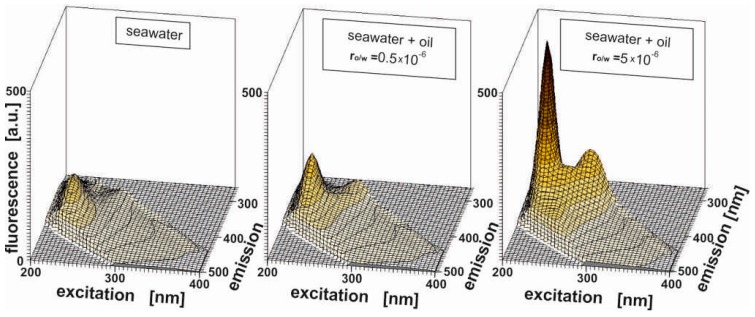
3D EEMs of natural seawater sample from the Southern Baltic Sea Gdynia-Orlowo walking pier and the seawater intentionally polluted by *Petrobaltic* crude oil for oil-to-water ratios of 0.5 × 10^−6^ and 5 × 10^−6^, respectively.

Furthermore, if the highest oil-to-water ratio (500 × 10^−6^) is considered, a third small peak is created and centered at 218 nm for an excitation wavelength corresponding to an emission wavelength of 300 nm. At the lower oil-to-water ratio (0.5 × 10^−6^), this peak is not sufficiently pronounced to form an actual peak. Moreover, it should be noted that the fluorescence of the major fluorescent peak described by Ex_max_/Em_max_ = 225/340 nm intensively increases in comparison with other determined peaks when the oil-to-water ratio increases, as displayed in Figure 3. Additionally, Figure 3 shows that the peak determined for seawater contaminated by oil partially overlaps the peak for original seawater. This fact makes the detection of oil pollution in original seawater difficult. Moreover, the spectra of seawater polluted by oil evolves when the oil concentration changes. Therefore, it is necessary to search for an efficient tool to detect oil in a quick and easy method.

### 3.2. Indicator of Oil Presence in the Sea Environment

**Figure 4:**
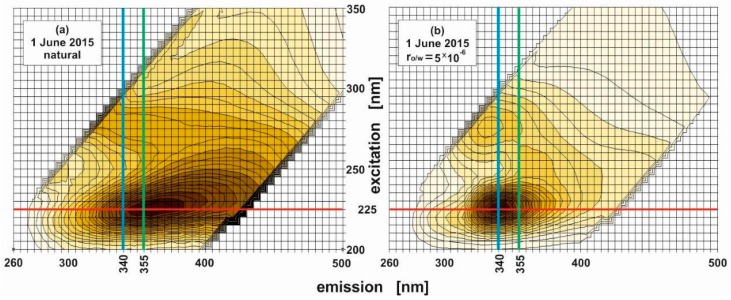
Natural seawater (**a**) and seawater exposed to oil pollution for an oil-to-water ratio of 5 × 10^−6^ (**b**). The red line indicates the chosen excitation wavelength, the green line shows the emission wavelength chosen for natural seawater and the blue line shows an emission wavelength chosen for seawater polluted by oil.

In view of the above findings, it is necessary to find an efficient indicator of the presence of oil substances in the water, assuming that these substances originate from pollution not necessary located strictly at the point of sampling, i.e., for example, they can be leached from oil residing in the vicinity of the sampling station. Based on the EEMs of dispersed oils in seawater and taking into account only seawater contaminated by oil only for one concentration of oil, Bugden [12] defined the parameter of the “intensity ratio” as the quotient of the fluorescence intensities for two defined emission wavelengths (corresponding to the detected emission maxima), consistent with the appropriate excitation wavelength. However, there is a need to detect oil pollution in the sea environment taking into account in situ measurement. Therefore, we are looking for an oil indicator extracted from EEMs of natural seawater and seawater polluted by oil. Considering the measured EEMs of natural seawater and seawater in contact with oil (examples are presented in Figure 4), it is seen that the main peaks for natural seawater and seawater contaminated by oil are detected for the same excitation wavelength centered at 225 nm (red line in Figure 4), whereas emission wavelengths for natural seawater and seawater polluted by oil are different; they are centered at 355 nm for natural seawater (green line in Figure 4) and centered at 340 nm for polluted seawater, regardless of the oil-to-water ratio (blue line in Figure 4).

This allows the fluorometric index (FI), introduced by Formula (1), to be expressed as a quotient of the fluorescence intensity at the emission wavelength for natural seawater to the intensity at the emission wavelength for seawater polluted by oil corresponding to the detected excitation maxima for both natural seawater and seawater polluted by oil. Therefore, in the FI definition we consider the natural seawater peaks with respect to peaks of seawater polluted by oil for various oil-to-water ratios.

(1) $$FI_{w/o}={\left[\frac{I\left(\lambda_{Emission\;of\;natural\;seawater}\right)}{I\left(\lambda_{Emission\;of\;seawater\;polluted\;by\;oil}\right)}\right]}_{\lambda_{Excitation}}$$

Furthermore, based on selected emission wavelengths for natural seawater (355 nm) and seawater polluted by oil (340 nm) corresponding to an excitation wavelength of 225 nm, the *FI_w/o_* was defined and computed for both natural seawater and seawater polluted by oil. For all oil-to-water ratios, *FI_w/o_* was calculated as the quotient of the fluorescence intensity at a 355 nm emission wavelength and an intensity at 340 nm, while the excitation wavelength remained equal to 225 nm (expressed by Formula (2)). For this calculation, the non-normalized data of the EEM spectra of natural seawater and seawater polluted by crude oil contour plots were used.

(2) $$FI_{w/o}={\left[\frac{I\left(\lambda_{Em=355}\right)}{I\left(\lambda_{Em=340}\right)}\right]}_{\lambda_{Ex=225}}$$

In the formula above, *I*(*λ_Em_*) describes, respectively, the fluorescence intensity corresponding to the emission wavelength for natural seawater (355 nm) and polluted seawater (340 nm) linked to the same excitation wavelength (*λ_Ex_*) for both kinds of seawater (225 nm).

The computed *FI_w/o_* index for natural seawater indicates values of approximately 1.1 (see Table 1), whereas the *FI_w/o_* for seawater contaminated by oil achieved values below 1, i.e., about 0.70 and about 0.73 for 1 June 2015 and 29 June 2015, respectively (see Table 2).

**Figure 5:**
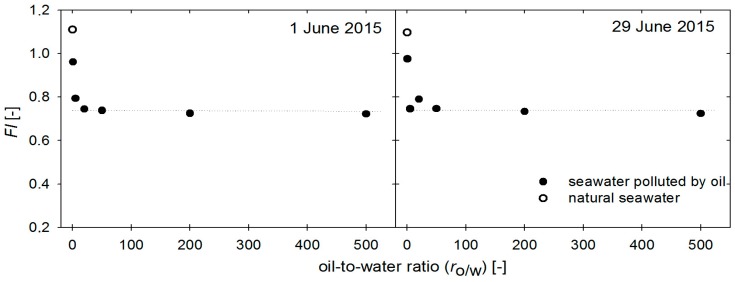
Fluorometric index (expressed by Formula (2)) as a function of oil-to-water ratio in seawater compared to the intensity ratio for the natural seawater.

Moreover, when interpreting the results for the *FI_w/o_* twice in June 2015, as shown in Figure 5, it should be noted that for seawater polluted by oil, the determined values of *FI_w/o_* are stable, regardless of the oil-to-water ratio for the studied cases. Moreover, Figure 5 shows the difference in FI values of natural seawater (white dot in Figure 5) and seawater polluted by oil for only a 0.5 × 10^−6^ oil-to-water ratio (black dots in Figure 5).

It should be stressed that the value of the emission wavelength (*λ_Em_* = 355 nm) for unpolluted seawater which was applied in the fluorometric index (Formula (2)) is a mean wavelength value for the maximum appearing in spectra of waters sampled 20 times in the spring and summer of 2015, after rejecting the extreme values. This matches the water for 29 June with an extreme value of 390 nm, although applying the principle of using 355 nm still turns out to be effective.

## 4. Conclusions

Based on the EEMs for natural seawater and the same water intentionally contaminated by very small amounts of oil, the fluorometric index (*FI_w/o_*) was determined by using the emission at 355 nm (maximal peak of fluorescence for natural seawater) and the emission at 340 nm (peak for seawater polluted with oil), while the excitation wavelength remained equal to 225 nm. The determined *FI_w/o_* for natural seawater indicates substantially higher values than that for seawater contaminated by oil. The results demonstrate that the *FI_w/o_* can be treated as an indicator to detect the presence of imperceptible oil, even when the oil pollution is not located at same place as the sampling point. Such a method would be particularly useful when oil is immersed below the sea surface or is emulsified (invisible to an above-water sensor). Furthermore, it is possible that, in this way, the appointed excitation and emission wavelengths could be useful to project a fluorometric sensor designed to detect oil appearing in the marine environment. Therefore, studies on defining the extent to which this method would be effective against a variety of refinery products will be continued. Taking into account the possibility of FI dependence on the type of seawater, sampling is planned in the future from both the open sea and other stations along the coast of the Baltic Sea.

## Figures and Tables

**Table 1:** Fluorescence index (*FI_w/o_*) calculated by Formula (2) for the uncontaminated seawater.

*FI_w/o_* [-]	
1 June 2015	29 June 2015
1.110	1.097

**Table 2:** Fluorescence index (*FI_w/o_*) calculated by Formula (2) for the seawater contaminated with oil.

r_o/w_	*FI_w/o_* [-]	
	1 June 2015	29 June 2015
0.5 × 10^−6^	0.751	0.915
5 × 10^−6^	0.721	0.727
20 × 10^−6^	0.705	0.757
50 × 10^−6^	0.705	0.730
200 × 10^−6^	0.707	0.722
500 × 10^−6^	0.709	0.718
